# Surgical Strategies for Siewert Type II Esophagogastric Junction Carcinomas: A Randomized Controlled Trial

**DOI:** 10.3389/fonc.2022.852594

**Published:** 2022-06-23

**Authors:** Kai Tao, Jianhong Dong, Songbing He, Yingying Xu, Fan Yang, Guolin Han, Masanobu Abe, Liang Zong

**Affiliations:** ^1^ Department of Gastrointestinal Surgery, Shanxi Cancer Hospital, Shanxi Medical University, Taiyuan, China; ^2^ Department of General Surgery, The First Affiliated Hospital of Soochow University, Suzhou, China; ^3^ Department of General Surgery, Yizhen People’s Hospital, Clinical Medical College, Yangzhou University, Yangzhou, China; ^4^ Department of Central Laboratory, Changzhi People’s Hospital, The Affiliated Hospital of Shanxi Medical University, Changzhi, China; ^5^ Department of Medical Records Room, Changzhi People’s Hospital, The Affiliated Hospital of Shanxi Medical University, Changzhi, China; ^6^ Graduate School of Medicine, University of Tokyo, Tokyo, Japan; ^7^ Department of Gastrointestinal Surgery, Changzhi People’s Hospital, The Affiliated Hospital of Shanxi Medical University, Changzhi, China

**Keywords:** Siewert type II esophagogastric junction carcinoma, proximal gastrectomy, total gastrectomy, jejunal interposition, esophagogastrostomy, Roux-en-Y Esophagojejunostomy

## Abstract

**Aim:**

To determine the ideal surgical approach for Siewert type II EGJ carcinomas.

**Methods:**

We conducted the randomized controlled trial (RCT) at Shanxi Cancer Hospital from January 2014 to August 2016. A total of 105 patients with T1-4N1-3M0 Siewert type II EGJ carcinomas were initially recruited. The final follow-up was up to June 30, 2019. Patients were randomized to undergo either a proximal gastrectomy plus jejunal interposition (PG+JI), proximal gastrectomy plus esophagogastrostomy (PG+EG), or total gastrectomy plus Roux-en-Y esophagojejunostomy (TG+RY). The primary endpoint was postoperative complications. Secondary endpoints were 5-year survival and recovery indexes.

**Results:**

Among 105 patients, 100 patients (95.2%; mean age, 56.2 years) with tumors <3cm in size underwent surgery: PG+JI (n=33) vs. PG+EG (n=33) and TG+RY (n=34); 91 patients completed the study. Among the groups, the PG+JI group had the longest reconstruction time: 34.11 ± 6.10 min vs. 21.97 ± 3.30 min (PG+EG) vs. 30.56 ± 4.26 min (TG+RY); p<0.001. There was no postoperative mortality. In the per-protocol analysis, the PG+JI group showed a decreased tendency in complication rate: 6.9% vs. 23.3% (PG+EG) vs. 18.8% (TG+RY), but there was no significant difference. For recovery indexes, the TG+RY group had the lowest values of the amount of single meal, weight loss, hemoglobin, albumin, pepsin, and gastrin among the three groups. There was no significant difference among the three groups in 5-year survival.

**Conclusions:**

Proximal gastrectomy is preferable for T1-4N1-3M0 Siewert type II EGJ carcinomas with tumors <3cm in size because of its better nutrition status under similar postoperative complication to total gastrectomy. Jejunal interposition can be recommended as a optional reconstruction approach after proximal gastrectomy.

**Clinical Trial Registration:**

https://www.chictr.org.cn/, identifier ChiCTR-IIR-16007733.

## Introduction

Esophagogastric junction (EGJ) carcinoma is defined as a tumor involving the junction between the esophagus and the stomach. Due to its rapidly increasing incidence, EGJ carcinoma has gained considerable attention in the last decades (1). The Siewert system classifies EGJ carcinomas into three subtypes based on the epicenter of the tumor, as follows. Type I: 1–5 cm above the junction, Type II: 1 cm proximal and 2 cm distal from the junction, and Type III: 2–5 cm distal from the junction (2). We submit that most surgeons would agree with the contention that Siewert type I EGJ carcinomas are best treated like distal esophageal cancer, and Siewert type III EGJ carcinomas are best treated like gastric cancer (3, 4). However, the mechanisms underlying the etiopathogenesis of type II EGJ carcinomas are not yet clear, and this type of EGJ carcinoma is thus the most controversial regarding the selection of surgical approaches (5).

Initially, transthoracic, transthoracoabdominal, and transhiatal esophagogastrectomy were all acceptable surgical approaches for Siewert type II EGJ carcinomas (6, 7). Siewert *et al.* speculated that most type II tumors are closer to proximal gastric cancer than distal esophageal adenocarcinoma, and they detected no significant difference in long-term survival between extended gastrectomy and esophagectomy (8). In addition, the results of the Japan Clinical Oncology Group (JCOG) 9502 RCT conducted in Japan demonstrated that the left thoracoabdominal approach cannot be justified to treat EGJ carcinomas, because of its increased morbidity and unimproved survival after the abdominal-transhiatal approach (TH) (9). It therefore seems that the abdominal-transhiatal approach is more advisable for Siewert type II EGJ carcinomas.

A retrospective analysis by Yamashita et al. showed that lymph node metastasis in Siewert type II EGJ carcinoma patients rarely occurred in no. 4, no. 5 and no. 6 lymph nodes regardless of whether the tumor’s epicenter was closer to the esophagus or the stomach (10). This finding suggested that a proximal gastrectomy might provide the same oncologic outcomes as those obtained with a total gastrectomy. However, esophagogastrostomy following a proximal gastrectomy has been reported to present the risk of severe reflux esophagitis (11). To improve the quality of life for patients who undergo a proximal gastrectomy, several reconstruction approaches have been devised, including jejunal interposition with a single or double tract (12). However, the optimal surgical approach for Siewert type II EGJ carcinomas has not been determined.

Here, to determine the ideal surgical approach for Siewert type II EGJ carcinomas, we conducted a randomized controlled trial to randomly assign patients to undergo a proximal gastrectomy plus jejunal interposition (PG+JI), a proximal gastrectomy plus esophagogastrostomy (PG+EG), or a total gastrectomy plus Roux-en-Y esophagojejunostomy (TG+RY).

## Patients and Methods

### Design and Patients

Between January 2014 and August 2016, we conducted a prospective RCT in Shanxi Cancer Hospital. The study was approved by the Institutional Review Board of Shanxi Medical University (**IRB File No. 2014-09-39**). All participants provided their written informed consent to participate in the study. An independent Data Safety Monitoring Committee reviewed the acquired data throughout the trial. The study was registered with the Chinese Clinical Trial Registry (No. ChiCTR-IIR-16007733) (http://www.chictr.org.cn/).

### Inclusion Criteria

The inclusion criteria required that patients (1) had no history of cancer, (2) had a documented diagnosis of EGJ carcinoma, (3) had a tumor <3 cm in size, (4) had no preoperative evidence of serosal invasion or distant extra-perigastric lymph node metastasis on preoperative CT scans, upper endoscopy, and endoscopic ultrasound, (5) had no serious comorbidity, and (6) had no metastasis in other organs during their surgery.

### Exclusion Criteria

We excluded patients with unstable angina or myocardial infarction within 6 months of the trial, severe respiratory disease, chronic renal disease, diabetes mellitus, or psychiatric disorders, as well as those found to have lymph node invasion or metastasis in other organs during surgery.

### Randomization and Blinding

We randomly assigned the patients at a 1:1:1 ratio to the PG+JI, PG+EG, and TG+RY groups. The Coordinating Center at Shanxi Medical University provided computer-generated random blocks of size 4 or 6. An independent masked nurse received the assignment and randomized the patients at the operating room. Although the surgeons could not be masked during the surgery, they were blinded during all postoperative follow-up.

Patients, caregivers, and outcome recorders were also blinded. The records detailing the surgical procedures were stored during the blinding period and were not available to any staff members until the completion of the study, unless there were postoperative complications.

### Interventions

Each PG+JI, PG+EG, or TG+RY was performed by one experienced surgeon, following standardized procedures (**Figure 2**). Importantly, this surgeon have finished 200 cases of PG+JI, 300 cases of PG+EG, or 3000 cases of TG+RY before this study. All patients underwent D2 plus No.19, No.20 and No.110 lymph node dissection. Postoperative patients with perigastric lymph node metastasis or a tumor penetrating the muscle layer of the gastric wall received six cycles of SOX chemotherapy.

### Reconstruction Methods

PG+JI, PG+EG, and TG+RY were performed as described previously (13, 14).

### Study Outcomes

The study’s primary endpoints were postoperative complications (Clavien-Dindo classification).

The study’s secondary endpoints were 5-year progression-free survival, overall survival, cumulative probability of recurrence and cumulative probability of mortality. We also determined the values of several nutrition indices: (1) the amount of single meal, (2) body weight, (3) albumin, (4) hemoglobin, (5) pepsin, (6) gastrin; and quality of life measures, i.e., (1) the classification of gastroesophageal reflux symptoms (Visick classification) (15), (2) the classification of endoscopic reflux symptoms (Los Angeles, LA) (16), and (3) the Gastrointestinal Symptom Rating Scale (GSRS) (13). The blood loss, the operative time, postoperative time to first flatus, length of hospitalization was also recorded.

### Statistical Analysis

The sample size calculation was based on the incidence of postoperative complications of our preliminary clinical study, in which the rate of PG+JI, PG+EG, TG+RY was 0, 30%, 10%, respectively (13, 14). We set the α= 0.05 and the β= 0.10. We calculated that the total sample size was 95 cases. Taking into account 20% of lost participants for various reasons, the number of cases in each group was increased to 119 cases.

The statistical significance of differences in the parameters were determined using Student’s t-test or Fisher’s exact test. Statistical significance was set at p<0.05. The SPSS 19.0 statistical package was used to perform all statistical analyses.

## Results

### Patient Characteristics

**Figure 1:**
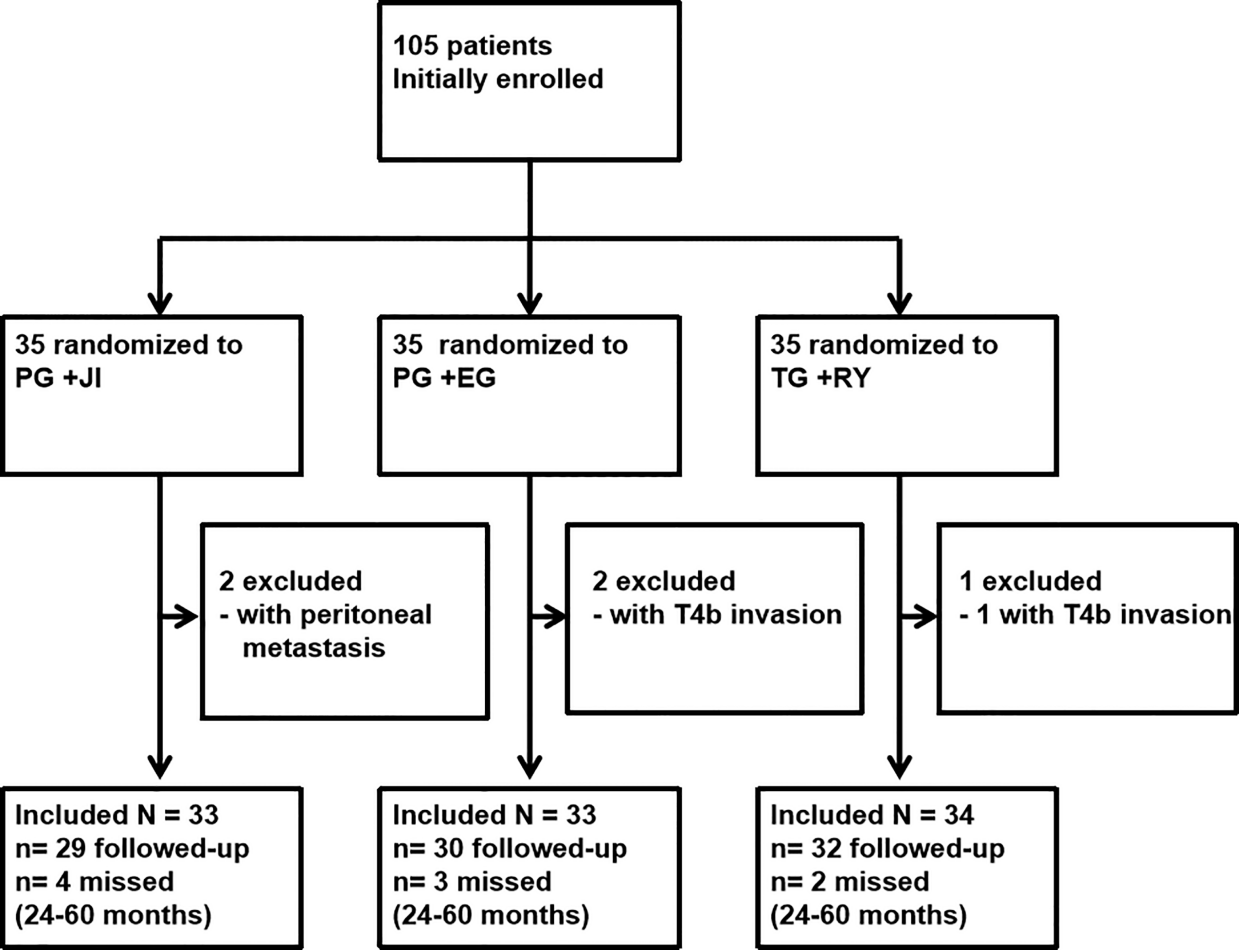
Trial Profile.

**Figure 2:**
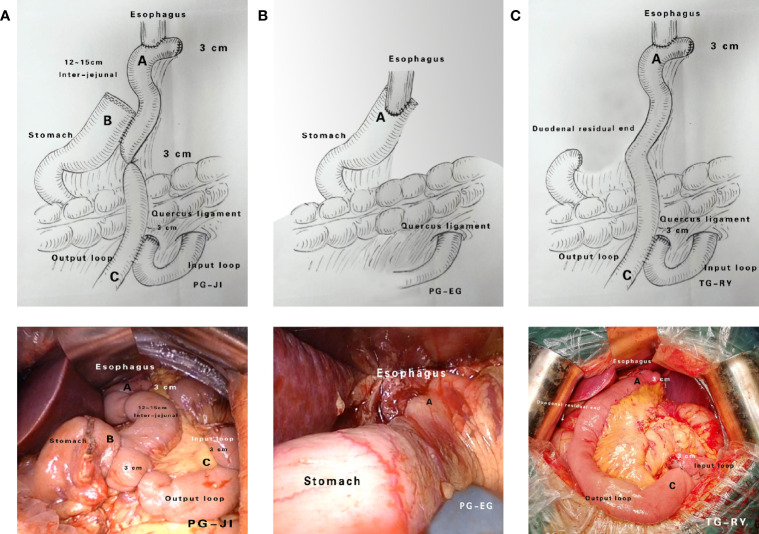
Surgical approaches. **(A)** Proximal gastrectomy + jejunal interposition (PG+JI). **(B)** Proximal gastrectomy + esophagogastrostomy (PG+EG). **(C)** Total gastrectomy + Roux-en-Y esophagojejunostomy (TG+RY).

From January 2014 to August 2016, we initially recruited 105 patients to undergo a PG+JI (n=35), a PG+EG (n=35), or a TG+RY (n=35) (**Figures 1**, **2**). 100 patients (95.2%; mean age, 56.2 years) with tumors <3cm in size underwent surgery: PG+JI (n=33) vs. PG+EG (n=33) and TG+RY (n=34); 91 patients completed the study (see **Figure 1**).

### Demographics

The three groups were similar with respect to age, gender, body mass index (BMI), and tumor-node-metastasis (TNM) stage (**Table 1**).

**Table 1:** Patient characteristics.

	PG+JI (n=29)	PG+EG (n=30)	TG+RY (n=32)	F /*X* ^2^	P value
Age (years)	60.36±8.14	60.70±7.11	59.63±6.66	0.165	0.685
Sex					
male	25 (86.2)	23 (76.7)	30 (93.8)	3.699	0.156
female	4 (13.8)	7 (23.3)	2 (6.3)		
BMI	23.84±1.91	23.85±1.77	23.50±2.28	0.469	0.495
pT				20.259	0.010*
Tis	0 (0)	1 (3.3)	0 (0)		
T1	9 (31.0)	2 (6.7)	1 (3.1)		
T2	5 (17.2)	8 (26.7)	3 (9.4)		
T3	4 (13.8)	9 (30.0)	8 (25.0)		
T4a	11 (37.9)	10 (33.3)	20 (62.5)		
pN				15.486	0.019*
N0	13 (44.8)	18 (60.0)	9 (28.1)		
N1	7 (24.1)	7 (23.3)	6 (18.8)		
N2	7 (24.1)	5 (16.7)	8 (25.0)		
N3	2 (6.9)	0	9 (28.1)		
Hemoglobin (g/L)	137.54±20.64	138.2±35.00	139.13±24.42	0.068	0.796
Albumin (g/L)	44.15±3.12	44.68±2.90	42.43±4.76	2.779	0.101

The meaning of the symbol "*" is that they have difference between groups.

### Clinical Outcomes

The PG+JI group had the longest reconstruction time among the three groups (p<0.001) (**Table 2**). The PG+JI and PG+EG procedures both took relatively less time for the first observation of intestinal peristalsis (p<0.001), and less length of hospital stay (1 (p=0.001), compared to the TG+RY group (**Table 2**). 

**Table 2:** Clinical outcomes.

	PG+JI (n=29)	PG+EG (n=30)	TG+RY (n=32)	F	P value
Reconstruction (minutes)	34.11±6.10	21.97±3.30^a^	30.56±4.26^ab^	52.92	0.000
Intestinal peristalsis (hours)	60.36±11.53	59.93±8.37	68.84±7.98^ab^	8.97	0.000
Hospitalization (days)	11.54±2.47	11.57±1.72	13.97±3.51^ab^	8.30	0.001

"a" means that there was significant difference with PG+JI group; "b" means that there was significant difference with PG+EG group (The same applies hereinafter).

### Postoperative Complications (Clavien-Dindo Classification)

There was no operative mortality in any of the groups. In the per-protocol analysis, the PG+JI group showed a decreased tendency in complication rate: PG+JI, 6.9%; PG+EG, 23.3%; TG+RY, 18.8%, but there was no significant difference (**Table 3**). In the TG+RY group, six patients experienced a postoperative complication: one with bleeding, two with dumping syndrome, and three with intestinal obstruction. For the patients with bleeding or intestinal obstruction, a conservative treatment was given.

**Table 3:** Postoperative complications (Clavien-Dindo classification).

	PG+JI (n=29)	PG+EG (n=30)	TG+RY (n=32)	*X* ^2^	P
I	1 (3.4)	2 (6.7)	2 (6.2)		
II	1 (3.4)	5 (16.7)	2 (6.2)		
IIIa	0	0	2 (6.2)		
IIIb	0	0	0		
IVa	0	0	0		
IVb	0	0	0		
Total	2 (6.9)	7 (23.3)	6 (18.8)	3.078	0.215

### Recovery Indexes

**Figure 3:**
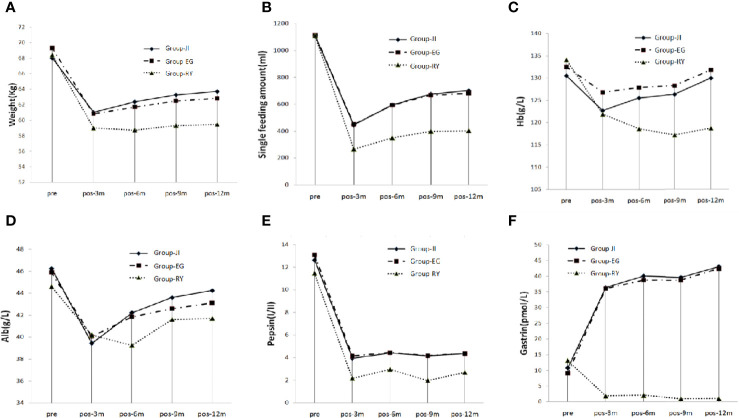
Nutrition Index. **(A)** Single food intake. **(B)** Body weight. **(C)** Hemoglobin. **(D)** Albumin. **(E)** Pepsin. **(F)** Gastrin.

The TG+RY group showed the lowest values of the amount of single meal, body weight, hemoglobin, albumin, pepsin, and gastrin among the three groups. At 3 months post-surgery, all the patients showed decreased tendency in the amount of single meal, body weight, hemoglobin, albumin and pepsin. However, a recovering tendency was observed at 6, 9, and 12 months post-surgery (**Figure 3**). At 3 months post-surgery, the gastrin level had fallen to nearly 0 in the TG+RY group, but it had increased by over three-fold in the two PG groups (**Figure 3**).

### Quality of Life Parameters

Visick classification: The PG+EG group’s percentage of scores over level II showed a increased tendency compared to the other two groups, and reached 26.6%, of which three cases were level III (**Table 4**). LA classification and GSRS score: The percentages of scores over A or 1 were higher in the PG+EG and TG+RY groups than that in the PG+JI group (**Table 4**).

**Table 4:** Postoperative symptoms.

	PG+JI (n=29)	PG+EG (n=30)	TG+RY (n=32)	F *X* ^2^	P value
Visiek classification					
I	27 (96.4)	22 (73.3)	27 (84.4)	6.840	0.314
II	1 (3.6)	4 (13.3)	3 (9.4)		
III	0 (0)	3 (10.0)	1 (3.1)		
IV	0 (0)	1 (3.3)	1 (3.1)		
LA classification					
0	20 (71.4)	12 (40.0)	11 (34.4)	15.314	0.020*
A	5 (17.9)	8 (26.7)	16 (50.0)		
B	3 (10.7)	7 (23.3)	4 (12.5)		
C	0 (0)	3 (10.0)	1 (3.1)		
GSRS score					
0	23 (82.1)	18 (60.0)	22 (68.8)	4.970	0.579
1	4 (14.3)	6 (20.0)	5 (15.6)		
2	1 (3.6)	4 (13.3)	4 (12.5)		
3	0 (0)	2 (6.7)	1 (3.1)		

The meaning of the symbol "*" is that they have difference between groups.

### Survival

**Figure 4:**
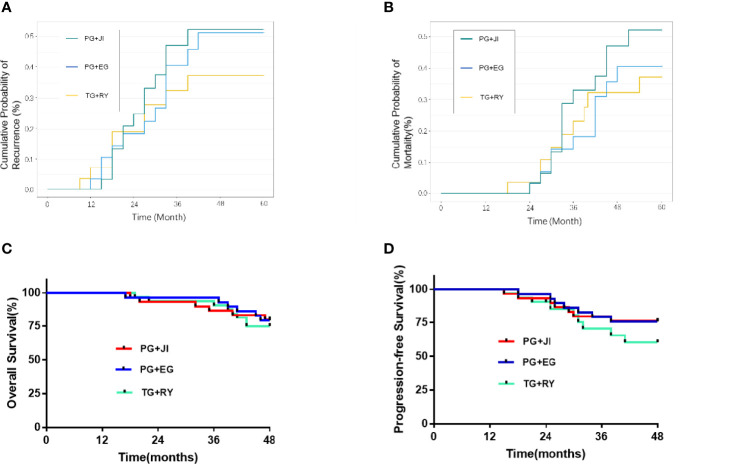
**(A)** Cumulative probability of recurrence. **(B)** Cumulative probability of mortality. **(C)** Overall survival. **(D)** 5-year progression-free survival.

There was no significant difference in the 5-year cumulative probability of recurrence among the three groups, or in the 5-year cumulative probability of survival (**Figure 4**).

## Discussion

In this series of patients with Siewert type II EGJ carcinomas, all three surgical procedures provided an R0 resection, and the postoperative data showed that none of the procedures resulted in serious morbidity or mortality, indicating the safety and feasibility of a proximal gastrectomy or total gastrectomy + D2 cleaning for T1-4N1-3M0 Siewert type II EGJ carcinomas. However, a proximal gastrectomy is more advisable because of its lower morbidity and its overall survival that is equivalent to that of total gastrectomy. In addition, our analysis of the study’s primary endpoints revealed quicker recovery of bowel function and shorter hospital stays after proximal gastrectomy. Although proximal gastrectomy+esophagogastrostomy posed a risk of serious reflux symptoms, it could be improved with JI reconstruction. Moreover, proximal gastrectomy showed clear superiority in the indices of postoperative nutrition and quality of life.

The National Comprehensive Cancer Network (NCCN) Clinical Practice Guidelines in Oncology-Gastric Cancer (ver. 3.2016) state that either a proximal gastrectomy or a total gastrectomy can be selected for patients with T1b-T3 proximal gastric cancer as long as a cut edge from the tumor can be >4 cm (17). The latest (2014) version of the Japanese Gastric Cancer Protocol suggests that jejunal interposition is an option after proximal gastrectomy (18). However, that protocol does not indicate the length of the inter-jejunum, or whether single-channel or dual-channel showed better anti-reflux effect.

The length of the jejunal limb is a very important issue that must be addressed when considering a JI reconstruction. Tokunaga et al. reported that a 10-cm or shorter length jejunal limb was recommended after PG (11). However, we suspect that such a short limb would not only cause a poor anti-reflux effect but also increase the incidence of anastomotic leakage because of high tension at the gastrointestinal anastomotic stoma. A 15-cm jejunal limb was therefore used in the present patient series. According to our data, this length did provide a sufficient anti-reflux effect, and only one patient (in the proximal gastrectomy+jejunal interposition group) developed reflux symptoms.

Regarding the postoperative nutrition indices, the the amount of single meal in the two PG groups was better than that in the TG group because of the maintenance of remnant stomach; therefore, the postoperative body weight and nutrition indexes were better in the PG groups than those in the TG group. We also observed that the average body weight of the PG+EG group was slightly higher than that of the PG+JI group before surgery, but it became slightly lower after surgery, suggesting that reflux symptoms might affect the postoperative diet. This may also explain the controversial result concerning body weight outcomes provided by different reconstruction approaches (19, 20).

The most important limitation of the present study is the small number of cases. Another issue is that the data were from only a single cancer center. A multi-center RCT is needed to test our findings. Siewert type II EGJ carcinoma is a very controversial tumor regarding both the resection extent and the reconstruction approach. Esophagectomy, gastrectomy, and left thoracoabdominal esophagogastrectomy, have strengths and weaknesses. Until better evidence is available the optimal approach should be tailored to the individual patient, and all three surgical options should be available at centers treating Siewert type II EGJ carcinoma (21). The results of our present analyses revealed similar oncologic outcomes between proximal gastrectomy and total gastrectomy. However, with an improved reconstruction method, i.e., jejunal interposition, proximal gastrectomy provided better short-term function and nutrition index results. For locally advanced Siewert type II EGJ carcinoma (cT3/4 or cN+), neoadjuvant chemotherapy followed by surgery is the standard approach (21).

In conclusion, this prospective RCT demonstrated that proximal gastrectomy is more advisable for T1-4N1-3M0 Siewert type II EGJ carcinomas because of its better nutrition status under similar postoperative complication to total gastrectomy, and our findings indicate that jejunal interposition can be recommended as a satisfactory reconstruction approach after proximal gastrectomy. Although our findings did confirm the feasibility and safety of performing PG+JI for T1-4N1-3M0 Siewert type II EGJ carcinomas, the use of jejunal interposition reconstruction required longer surgery times than esophagogastrostomy.

## Data Availability Statement

The original contributions presented in the study are included in the article/supplementary material. Further inquiries can be directed to the corresponding author.

## Ethics Statement

The studies involving human participants were reviewed and approved by Shanxi Medical University. The patients/participants provided their written informed consent to participate in this study.

## Author Contributions

LZ contributed to the study conception and design. Material preparation, data collection and analysis were performed by SH, MA,YX, FY, and GH. The first draft of the manuscript was written by KT, JD, and all authors commented on previous versions of the manuscript. LZ, GH, SH, and KT revised the manuscript. All authors contributed to the article and approved the submitted version.

## Funding

Wu Jieping Medical Foundation (Grant No. 320.6750.2020-11-5), Health commission of Shanxi Province (Grant No. 2020130), Natural Science Foundation of Shanxi Province (Grant No. 202103021224005), Excellent Overseas Students Project Fund of Shanxi Province(Grant No. 20220056), Project of Gusu Medical Key Talent of Suzhou City of China (Grant No. GSWS2020005), and the Project of New Pharmaceutics and Medical Apparatuses of Suzhou City of China (Grant No. SLJ2021007).

## Conflict of Interest

The authors declare that the research was conducted in the absence of any commercial or financial relationships that could be construed as a potential conflict of interest.

## Publisher’s Note

All claims expressed in this article are solely those of the authors and do not necessarily represent those of their affiliated organizations, or those of the publisher, the editors and the reviewers. Any product that may be evaluated in this article, or claim that may be made by its manufacturer, is not guaranteed or endorsed by the publisher.
